# Evaluating the impact of a structured medication optimisation review on prescribing patterns and bleeding risk among patients prescribed direct oral anticoagulants (DOACs): a difference-in-differences study

**DOI:** 10.1136/bmjoq-2025-003568

**Published:** 2025-11-21

**Authors:** Elizabeth M Camacho, Olly Butters, Konstantinos Daras, Jennifer Downing, Joanne Bateman, Susanne Lynch, Iain Edward Buchan, Benjamin Barr

**Affiliations:** 1 Institute of Population Health, University of Liverpool, Liverpool, UK; 2 Countess of Chester Hospital NHS Foundation Trust, Chester, England, UK; 3 NHS Cheshire and Merseyside Integrated Care Board, Warrington, England, UK

**Keywords:** Medication safety, Adverse events, epidemiology and detection, Decision support, clinical

## Abstract

**Objective:**

To evaluate the safety of implementing structured medication optimisation reviews (SMORs) for patients with atrial fibrillation (AF) prescribed direct oral anticoagulants (DOACs). SMORs aimed to improve quality of care and facilitate large-scale alignment with national prescribing guidance (to switch patients onto edoxaban).

**Intervention:**

Complex intervention including a SMOR embedded within primary care electronic patient records alongside clinical decision support tools.

**Design:**

Doubly robust difference-in-difference analysis using linked electronic health records, comparing changes in prescribing and bleeding admissions in patients undergoing SMOR with those in patients not reviewed.

**Setting:**

Sefton (intervention area) and Liverpool (comparator area) in the Northwest of England.

**Participants:**

All patients with AF prescribed a DOAC in 2022.

**Main outcomes and measures:**

Proportion of patients prescribed apixaban, proportion of patients prescribed edoxaban and rate of emergency hospital admissions for bleeding-related events.

**Results:**

The proportion of patients in Sefton prescribed edoxaban increased from 19% to 35%; 13% (95% CI 11% to 14%) of the increase was associated with the SMOR. There was an 11-percentage point decrease in patients prescribed apixaban (95% CI −12% to −10%). Undergoing review was associated with a non-significant reduction in the risk of bleeding admissions (eight fewer admissions per 1000 people reviewed per year; 95% CI −22 to 6).

**Conclusions:**

SMORs can be delivered at scale and used to switch medications for a large proportion of people. There was no evidence of an increased risk of admissions for bleeding complications in AF patients following a large-scale switch from apixaban to edoxaban supported by SMORs. Such reviews could improve prescribing quality and patient safety by ensuring patients are on the most appropriate dose and choice of DOAC and lead to cost savings to health services (by facilitating a switch to a better value product) while not increasing risks for patients.

**WHAT IS ALREADY KNOWN ON THIS TOPIC**

- Atrial fibrillation (AF) is estimated to cost the National Health Service (NHS) in England between £1.4 billion and £2.6 billion per year.
- Direct oral anticoagulants (DOACs) can reduce the risk of stroke in people with AF but increase the risk of bleeds—regular medication reviews can mitigate this risk.

**WHAT THIS STUDY ADDS**

- Using routinely collected patient-level data, this study found that structured medication optimisation reviews (SMORs) could be used as part of a large-scale programme to switch patients to a less expensive DOAC.
- The SMOR programme did not compromise (and may improve) safety in relation to adverse bleeding events.

**HOW THIS STUDY MIGHT AFFECT RESEARCH, PRACTICE OR POLICY**

- This study provides evidence of a safe and effective SMOR programme that commissioners could implement to facilitate alignment with national prescribing policy and reduce prescribing costs.

## Introduction

 Atrial fibrillation (AF) affects around 2% of the population of England.^1^ Prevalence is rising due to the increasing age of the population and survival of people with AF.^2^ It is estimated to cost the National Health Service (NHS) between £1.4 billion and £2.6 billion per year—between 0.9% and 1.6% of total NHS expenditure.^2^ It is an important risk factor for thromboembolism and stroke. Patients with a diagnosis of AF are prescribed an anticoagulant to reduce this risk. From 2013, a new group of anticoagulants, direct oral anticoagulants (DOACs), was introduced, including dabigatran, rivaroxaban, apixaban and edoxaban,^3^ with the National Instititue for Health and Care Excellence (NICE) recommending their use for stroke prevention in AF. Unlike the warfarin therapy they were intended to replace, DOACs did not require as many blood tests to monitor therapy, which are inconvenient for the patient and use NHS resources. In 2019, the NHS set a target to treat 90% of AF patients with DOACs and this target was met in 2021.^4^ While there are clear benefits from this leading to a reduction in haemorrhagic stroke, like all anticoagulants, DOACs are associated with increased risk of bleeding and are relatively expensive medicines. Anticoagulation prescription costs in England rose from £87 million in 2011–2014 to £769 million in 2014–2017 due to the introduction of DOACs.^5^

In 2022, NHS England established an agreement with the manufacturers of edoxaban leading to it being offered to the NHS at a lower price than the other most frequently prescribed DOAC apixaban.^6^ As a result of it becoming the most cost-effective option, clinicians in the NHS were recommended and incentivised (via NHS England’s ‘Investment and Impact Fund’)^7^ to prescribe edoxaban rather than other more expensive DOACs where clinically appropriate. This included switching patients onto edoxaban from other DOACs, usually apixaban. In 2022, the then Clinical Commissioning Group (CCG) medicines management team in Sefton, Northwest England, began carrying out structured medication optimisation reviews (SMORs) on patients with AF currently receiving a DOAC. This was in line with a regional project to improve the quality of DOAC prescribing (thereby improving patient safety through assessment of risk and appropriate modification of prescription). The SMORs were undertaken to identify if patients were on an appropriate DOAC and dose as well as checking compliance and identifying adverse reactions to DOACs. Patients were then identified to be switched to edoxaban where it was clinically appropriate and safe to do so.

There have been no previous studies, to our knowledge, assessing the impact and potential bleeding risks of using such an approach to switch patients from one DOAC to another. Such a review and switching process could directly benefit patients if the new medication is prescribed at a more clinically appropriate dose or is safer. It could however be harmful if the new medication is less beneficial or if changing the patient’s medication leads to an adverse drug event. We therefore evaluated the impact of introducing SMORs for AF patients prescribed a DOAC in Sefton using a difference-in-differences study design to understand the causal impact of the programme on DOAC prescribing patterns and admissions for bleeding disorders.

## Methods

### Data and measures

We used anonymised linked data from primary care and hospitals for all patients registered with a primary care (general practitioner (GP)) practice in Sefton and Liverpool that had signed a data sharing agreement (98% of practices). Sefton is an area in Northwest England where the SMOR intervention was implemented. Sefton is a neighbouring district to Liverpool where no similar intervention was implemented during the study period. We included data from Liverpool to provide a larger pool of potential ‘controls’ (ie, patients who did not receive a SMOR). Patients were included in the cohort if they had a diagnosis of AF recorded in their electronic GP record before 1 January 2022 and had a prescription for apixaban or edoxaban as of 1 January 2022. We also included AF patients with a prescription for other DOACs so that we could identify if other changes in prescribing patterns occurred during the evaluation period (see online supplemental appendix 1 for specific doses and clinical codes for each DOAC included). We excluded DOACs/doses not licenced for monotherapy.

We assessed the impact of the intervention on two outcomes. First, to investigate the impact on alignment of prescribing with national guidance, we explored the proportion of AF patients with a recorded prescription of apixaban or edoxaban. To do this, the specific DOAC that patients were prescribed was flagged within each quarter of the calendar years 2021–2023. Second, we looked at the number of emergency hospital admissions for a bleeding-related disorder or a clotting-related disorder in each quarter of the calendar years 2021–2023. A full list of International Classification of Diseases, Tenth Revision (ICD-10) codes used to define these hospital admissions is reported in supplementary materials (online supplemental appendix 2).

To account for potential differences in morbidity and disease severity between those reviewed and not reviewed that could influence these outcomes (ie, confounding factors), we considered several other variables. Demographic characteristics included age, sex, ethnic group and Index of Multiple Deprivation (IMD). IMD is a composite measure indicating the level of poverty within the neighbourhood in which people live—a proxy measure of their socioeconomic circumstances.^8^ Clinical factors included time since initial AF diagnosis, time since first DOAC prescription, diagnosis of comorbid conditions (coronary heart disease, chronic kidney disease (CKD), diabetes, cancer, chronic liver disease and heart failure), number of emergency hospital admissions for clotting, bleeding disorders and admissions from any other cause in 2021 (ie, pre-dating the implementation of the SMOR programme).

### Intervention

The intervention was a SMOR using an electronic template and clinical decision support tools which included decision aids for initiating and reviewing patients on DOACs and a standard operating procedure (see online supplemental appendix 3 for copies of these documents). To support the implementation of the SMOR programme, two educational webinars were run by clinical experts to increase clinical knowledge and talk through use of the template. The webinars were attended by pharmacists (primarily based in primary care), medicines management leads, pharmacy technicians and GPs.

The reviews started at the end of 2021 and rapidly increased in frequency through 2022 before levelling off by the end of the first quarter of 2023 (see online supplemental appendix 4). The SMOR involved completing a clinical electronic template specifically designed for this project which was embedded within the primary care electronic patient record system (EMIS). The template automatically incorporated clinical information and facilitated consistent and efficient assessment of the patient’s history and risk of stroke (CHA₂DS₂-VASc score^9^) or bleeding (HAS-BLED^10^ or ORBIT^11^ score) and a review of the patient’s weight, history of gastrointestinal symptoms and current renal function (including calculation of the creatinine clearance). The decision aids ask reviewers to check if patients have previously changed from an alternative DOAC due to intolerance or treatment failure and to assess for potential interacting medications, swallowing ability (ie, if tablets are required to be crushed), adverse reactions and compliance. This information was then used to assess if it was clinically appropriate to switch the patient to edoxaban. The reviews were conducted typically by a pharmacist in a primary care setting (although in some practices it would be a GP) at one-to-one appointments, either face-to-face (at patient’s usual primary care practice) or over the telephone. Once all test results had been reviewed, if medication changes were required or recommended, the patient was contacted and changes agreed.

Completion of the template was recorded in electronic patient records with an agreed set of codes (see online supplemental appendix 5 for details of the codes used). For this analysis, the date of review was taken as the earliest date after 1 January 2022 when one of the DOAC review codes was entered in the patient’s electronic record. The intervention group was defined as all patients in Sefton who were recorded as having received a SMOR during 2022 or 2023.

### Control

The control group was all other AF patients in the study cohort in Sefton who were not recorded as having received the intervention as defined above and AF patients in Liverpool (where the SMOR programme was not implemented). Any AF patients in Liverpool who were recorded as having had a SMOR were excluded from the analysis. For control patients, we imputed a placebo review date such that the distribution of the placebo reviews across calendar time was the same as for the intervention group.

### Analysis

Analyses were conducted in R (R V.4.3.0, http://www.r-project.org). To estimate the average effect of the review on outcomes, we use difference-in-differences with a doubly robust estimation procedure developed by Sant’Anna and Zhao.^12^ Difference-in-difference methods compare the average change in an outcome over time in an intervention group to the equivalent average change over time for a control group. The difference in these changes in outcome between the intervention and control groups can be taken as the average treatment effect on the treated of the intervention, if a set of assumptions holds, the most important being the parallel trends assumption.^13^ The parallel trends assumption requires that in the absence of the intervention, the difference between the intervention group and the control group is constant over time. To initially visualise the trends in the data, we plotted the outcomes before and after review in the intervention and control populations.

While it is not possible to be certain whether the assumptions hold, we can make it more plausible by conditioning on pre-intervention covariates to balance the intervention group and the control group in terms of characteristics that might be related to the outcome. The doubly robust estimation method achieves this using inverse probability tilting to estimate the conditional propensity score of being treated, essentially creating a weighted population where observed covariates are balanced between the intervention and control populations. We included all the control variables outlined above in this regression model to account for these observed differences between the intervention and control groups. These weights are then used in weighted least squares regressions for the difference in the evolution of the outcome in the intervention group compared with the control group. This approach allows for treatment effects to vary by timing of review and by quarter following review. We use this model to estimate the average treatment effect over the whole 2022–2023 period. We also plot dynamic treatment effects—that is, showing how the effect of the review on bleeding-related admissions changes over follow-up time and testing whether trends are conditionally parallel prior to the intervention, providing some assurance as to whether the parallel trends assumption is met (conditional on the observed controls).

### Sensitivity analyses

To confirm the robustness of our findings, we conducted several sensitivity analyses. As selection into the control group from the non-reviewed in Sefton could bias results, but also populations in Liverpool could have been affected by unobserved influences that were not present in Sefton, we re-estimated the overall effect size on bleeding admissions using alternative control populations: (1) just people from Liverpool and (2) just people from Sefton who had not received a SMOR. We additionally estimated another model using inverse probability weighting and ordinary least squares, rather than inverse probability tilting, and using only ‘never treated’ (ie, never had a SMOR) individuals as controls.

### Patient involvement

This study involved the secondary use of existing data sources and did not include patients as study participants. No patients were involved in setting the research question, the study design or the overall conduct of the study. Patients and patient representatives within the National Institute for Health Research, Applied Research Collaboration North-West Coast (ARC NWC) will be supported to write a lay language summary, and the results of the study are going to be disseminated to the relevant patient and public groups through the ARC NWC communication programme.

## Results

In total, across Sefton and Liverpool, there were 10 760 people with AF prescribed a DOAC at the beginning of 2022 when the SMORs started. 2685 patients in Sefton were coded as having had a structured review during 2022 or 2023. The characteristics of the sample are reported in table 1. Those experiencing a SMOR were less likely to have CKD, heart failure and diabetes; live in a deprived area; have been prescribed edoxaban before review and have been admitted to hospital in the past year. They were more likely to have been prescribed apixaban and were slightly older. All these factors were controlled for in the analysis and should not bias our results.

**Table 1:** Characteristics of the intervention and control cohort at the beginning of 2022

	Control	Intervention	P
n	8075	2685	
Number of months receiving a direct oral anticoagulant (DOAC) (mean (SD))	38.60 (25.23)	38.59 (24.72)	0.980
Number of months since diagnosis with atrial fibrillation (mean (SD))	62.42 (51.58)	64.46 (53.78)	0.079
Chronic kidney disease (%)	41	37	0.001
Heart failure (%)	93	91	0.032
Chronic liver disease (%)	5	5	0.368
Cancer (%)	24	26	0.066
Diabetes (%)	27	24	0.001
Index of Multiple Deprivation (mean (SD))	36 (22)	23 (17)	<0.001
Age (mean (SD))	77 (10.6)	78 (9.4)	<0.001
Male sex (%)	56	56	0.688
DOAC initially prescribed (%)			<0.001
Apixaban	59	70	
Dabigatran	3	3	
Edoxaban	27	19	
Rivaroxaban	11	8.3	
Patients with at least one hospital admission in 2021 (%)	46	43	0.006
Number of admissions from all causes in 2021 (mean (SD))	0.99 (1.69)	0.88 (1.65)	0.002

**Figure 1:**
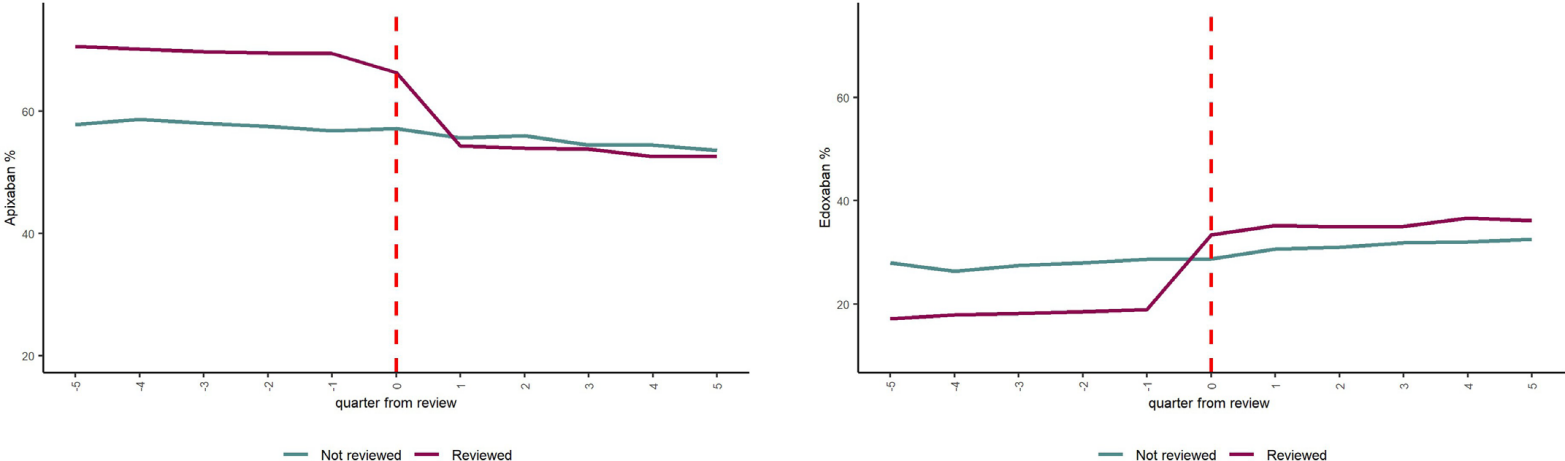
Percentage of patients prescribed edoxaban (left) or apixaban (right) by quarter since review.

Figure 1 shows the proportion of AF patients in Sefton who were prescribed apixaban (left panel) and edoxaban (right panel) among those who received a structured review compared with those who did in the quarters before and after review. We see a marked increase in the proportion prescribed edoxaban and a marked decrease in the proportion prescribed apixaban immediately following the review in the intervention group and no equivalent change in the control group. In the quarter following their SMOR, 35.2% of patients were prescribed edoxaban (compared with 30.1% of control patients in the quarter after their placebo SMOR date).

**Figure 2:**
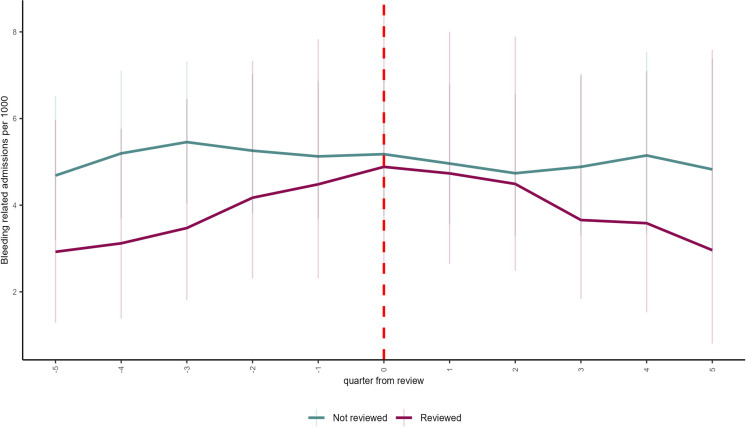
Admissions per 1000 people by quarter since review.

Figure 2 shows the rate of admissions for bleeding complications in the quarters before and after the review. There were 395 admissions for bleeding complications across the intervention (89 admissions) and control (306 admissions) cohorts during this period. The trends in both groups are not markedly different. There was a slight increase in admissions in the intervention group leading up to the review and a slight decline afterward. There were 39 admissions for clotting disorders overall during the study period, with only 5 in the reviewed group (and 34 in the non-reviewed group). Due to the small number of clotting-related events in the data, we have not reported any additional analysis for this outcome.

Table 2 shows the overall results from the doubly robust difference-in-differences model indicating the average treatment effect on the treated for each of our outcomes, that is, the change in outcomes attributable to the intervention. Undergoing review led to an additional 12.8% (95% CI 11.3% to 14.2%) of patients reviewed being prescribed edoxaban and a reduction of 11.0% of patients reviewed being prescribed apixaban (95% CI −12.3% to −9.7%). Overall, the review was associated with a non-significant reduction of eight bleeding admissions per 1000 patients reviewed per year of follow-up (95% CI −22.2 to 6.1).

**Table 2:** Change in outcomes attributable to the intervention estimated from the doubly robust difference in differences model

Outcome	Estimate	95% CI	
Edoxaban (percentage point change)	12.8%	11.3%	14.2%
Apixaban (percentage point change)	−11.0%	−12.3%	−9.7%
Bleeding related admissions (change per 1000 reviewed per year)	−8.1	−22.2	6.1

**Figure 3:**
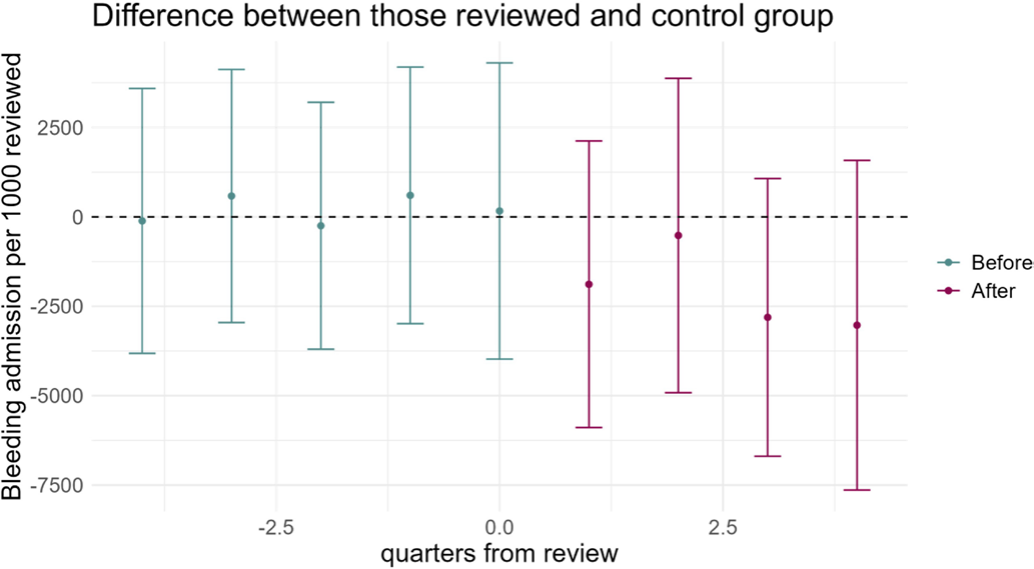
Difference in number of bleeding admissions for those receiving a structured medication optimisation review and controls in the quarter before and after the review.

Figure 3 shows the quarterly estimates from the doubly robust difference-in-differences model, that is, the estimated difference in the quarterly rate of emergency hospital admissions in people who did and did not have a DOAC review by time since the review date. This indicates any change in effect, depending on the time since review, as well as a check of the parallel trend assumption. There is some indication that the review may have led to lower risk of bleeding admissions, over longer periods of follow-up, but these are not statistically different from zero so may reflect random fluctuations. Prior to the review, there was no difference in risk of bleeding admissions between those who were never reviewed and those who subsequently experienced a structured review indicating that trends were approximately parallel (p=0.23). Sensitivity analysis using alternative control groups and inverse probability weighting showed similar results (see online supplemental appendix 6).

## Discussion

We found that SMORs increased the proportion of patients with AF whose medication was switched from apixaban to edoxaban (in line with national guidance at the time) and there was no evidence that switching patients from apixaban to edoxaban was associated with bleeding-related harm (ie, there was a non-significant reduction in bleeding risk associated with having a SMOR).

Regular medication reviews are important for AF patients on anticoagulant therapy, including monitoring stroke and bleeding risk and modifying doses accordingly. The European Heart Rhythm Association Guidelines recommend regular monitoring of AF patients^14^ and NHS guidance recommends that all patients prescribed a DOAC should have at least an annual medicine optimisation review.^15^ However, medicine optimisation reviews are not consistently happening in the UK and there are no robust systems in place to ensure patients taking a DOAC are reviewed. In addition to improving safety, better monitoring also provides an opportunity to optimise resource uses.

There have been no randomised trials directly comparing the safety of apixaban and edoxaban. Evidence instead has come from comparative reviews and observational studies which paint a mixed picture. One systematic review of network analyses found that although all 13 analyses found apixaban was associated with lower risk of major bleeds compared with edoxaban, none showed a statistically significant difference.^16^ An inverse probability (of treatment) weighted comparison of 21 000 patients prescribed DOACs found no difference in risk of major bleeds between apixaban and edoxaban.^17^ A similar observational electronic healthcare databases study of 527 000 new DOAC users in France, Germany, the UK and the USA reported that apixaban was associated with a significantly lower risk of gastrointestinal bleeding than edoxaban.^18^ Another study in Belgium of 250 000 patients exposed to DOACs using inverse probability (of treatment) weighting found apixaban had a significantly lower major bleeding risk compared with edoxaban.^19^ These observational studies may be biased due to unobservable characteristics of patients that may influence clinician and patient prescribing decisions and choices that are also associated with differences in bleeding risk. The programme of switching patients from apixaban to edoxaban implemented in the UK provides a more exogenous source of variation in prescribing pattern that will be less affected by these biases.

There are several strengths and limitations to our analysis. Linkage of primary and secondary care data and consistent coding of SMOR enabled robust causal methods to be applied in estimating the impact of medicine reviews on an important safety outcome. This enabled our study to take account of both observed and unobserved time-invariant confounders and differential trends that were related to baseline observed differences in characteristics between intervention and control groups. Additionally, as undertaking reviews during this time was likely a more exogenous source of variation in prescribing than changes in prescribing during routine clinical practice, our study represents a less biased estimate of the effect of switching from edoxaban to apixaban than previous observational studies. It is possible that dose correction as a result of the SMOR process, in addition to switching DOACs, is related to bleeding events. However, within the electronic records analysed, it is not possible to categorically determine whether or not a particular DOAC prescription is for an appropriate dose and so we are unable to explore this further.

Although our study has a reasonably large sample size, admissions for bleeding are relatively infrequent; consequently, there remains considerable uncertainty around our estimates. Studies relying on electronic health records are dependent on the accuracy and consistency of clinical coding. Clinicians undertaking the reviews were instructed on codes to apply and numbers recorded on the clinical system were compared with aggregate records maintained by medicines management teams. Although the combinations of codes were likely to be specific to the structured medicine optimisation review, it was also possible for other primary care staff to use those codes for other non-structured reviews. Some people receiving the intervention therefore may have been missed if not coded and some may have been coded as receiving the intervention when they did not. The clear discontinuity in changing prescribing at the review date however indicates that in most cases the coded intervention point was accurate. It is possible that some people in the control group received a DOAC review which was not sufficiently structured to be coded as a SMOR in their health records. However, our aim was to evaluate the effectiveness of the structured review specifically; therefore, this does not change the interpretation of our findings. Some people in the control area (Liverpool) had a code in their records to indicate that they did receive a structured review. However, this was a very small number of people (n=191; 3% of total sample from Liverpool), and they were excluded from the analysis. Our sensitivity analysis which only included as controls people in the intervention area (Sefton) who did not receive a SMOR showed similar results to the primary analysis.

The outcome we used included a broad definition of bleeding-related admissions we had used in a previous study,^3^ in order to be inclusive of all admissions that could be affected by increased bleeding risk. Focusing on more severe bleeds may have been more specific, although these bleeds were all severe enough to warrant admission. As our outcome only included admissions, we were not able to assess the impact on less severe bleeding. We explored clotting-related admissions (including cerebral infarction, stroke, pulmonary embolism and deep vein thrombosis). However, there were too few events to allow any meaningful inference about the impact of SMORs (only five events were recorded in people who received a SMOR).

The findings have important implications for practice. Switching medications to equally safe and effective, but cheaper, alternatives is an important way in which health systems can minimise expenditure, enabling greater investment in other effective measures, increasing net benefits on a fixed budget. It is vital that strategies like the SMOR programme can be deployed to react to policy changes. For example, in September 2024, NHS England revised their guidance on the prescribing of DOACs for AF patients in response to the expiration of the patent on apixaban and rivaroxaban.^20^ This opened the door for the development of generic versions of these DOACs, meaning that they became the DOACs with the lowest acquisition cost. The guidance was that AF patients newly initiated on a DOAC should be prescribed one of these options. In addition, commissioners were advised to develop local programmes to ‘review’ AF patients already established on a DOAC. It is acknowledged that while the specific switch from apixaban to edoxaban explored in this study no longer aligns with current prescribing policy, our findings still have application beyond this. We have provided evidence about a programme to improve patient safety involving a high-risk medication that commissioners could implement to meet the new guidance. The programme involved implementing a system-wide robust, consistent, evidence-based approach to review DOAC treatment and provide appropriate training and clinical support to facilitate primary care pharmacists and GPs to deliver it. A key concern was that switching high-risk drugs such as DOACs puts the patient at a higher risk of adverse events and this study has highlighted that this has not been seen. Therefore, should further switches need to be considered, for example, from edoxaban to cheaper DOACs, it provides some evidence that this is safe.

## Supplementary material

10.1136/bmjoq-2025-003568 online supplemental file 1

## Data Availability

No data are available.
