# Supplementary material for: Evaluating the impact of a structured medication optimisation review on prescribing patterns and bleeding risk among patients prescribed direct oral anticoagulants (DOACs): a difference-in-differences study
Source: BMJ Open Qual. 2025 Nov 21;14(4):e003568. doi: 10.1136/bmjoq-2025-003568 (PMC12658506; doi:10.1136/bmjoq-2025-003568)
Supplement: online supplemental file 1 [file bmjoq-14-4-s001.docx]

**APPENDICES: Evaluating the impact of a structured medication optimisation review on prescribing patterns and bleeding risk among patients prescribed direct oral anticoagulants (DOACs). A difference-in-differences study.**

**Appendix 1. SNOMED and BNF codes for DOACs included in the analysis**

- Apixaban 2.5mg - (703907006, 0208020Z0AAAAAA)
- Apixaban 5mg – (703908001, 0208020Z0AAABAB)
- Dabigatran 110mg – (13532811000001109, 0208020X0AAABAB)
- Dabigatran 150mg – (19469811000001101, 0208020X0AAACAC)
- Edoxaban 30mg – (29903311000001108, 0208020AAAAABAB)
- Edoxaban 60mg – (29903411000001101, 0208020AAAAACAC)
- Rivaroxaban 20mg – (19842211000001107, 0208020Y0AAACAC)
- Rivaroxaban 15mg - (19842111000001101, 0208020Y0AAABAB)

Note that DOACs are excluded from this list (e.g. Edoxaban 15mg (29903211000001100) that are not licenced doses for monotherapy).

**Appendix 2. ICD-10 codes used to define hospital admissions**

*Bleeding-related admissions*

M79.81 traumatic haematoma of the soft tissue

M25.00 Hemarthrosis, unspecified joint

K92 Other diseases of digestive system

K92.0 Haematemesis

K92.1 Melaena

K92.2 Gastrointestinal haemorrhage, unspecified

K62.5 Haemorrhage of anus and rectum

R58 Haemorrhage, not elsewhere classified

R04.0 Epistaxis

R04.1 Haemorrhage from throat

R04.2 Haemoptysis

R04.8 Haemorrhage from other sites in respiratory passages

R04.9 Haemorrhage from respiratory passages, unspecified

R31 Unspecified haematuria

N02 Recurrent or persistent haematuria

I60 Subarachnoid haemorrhage

I60.0 Subarachnoid haemorrhage from carotid siphon and bifurcation

I60.1 Subarachnoid haemorrhage from middle cerebral artery

I60.2 Subarachnoid haemorrhage from anterior communicating artery

I60.3 Subarachnoid haemorrhage from posterior communicating artery

I60.4 Subarachnoid haemorrhage from basilar artery

I60.5 Subarachnoid haemorrhage from vertebral artery

I60.6 Subarachnoid haemorrhage from other intracranial arteries

I60.7 Subarachnoid haemorrhage from intracranial artery, unspecified

I60.8 Other subarachnoid haemorrhage

I60.9 Subarachnoid haemorrhage, unspecified

I61 Intracerebral haemorrhage

I61.0 Intracerebral haemorrhage in hemisphere, subcortical

I61.1 Intracerebral haemorrhage in hemisphere, cortical

I61.2 Intracerebral haemorrhage in hemisphere, unspecified

I61.3 Intracerebral haemorrhage in brain stem

I61.4 Intracerebral haemorrhage in cerebellum

I61.5 Intracerebral haemorrhage, intraventricular

I61.6 Intracerebral haemorrhage, multiple localized

I61.8 Other intracerebral haemorrhage

I61.9 Intracerebral haemorrhage, unspecified

I62 Other nontraumatic intracranial haemorrhage

I62.0 Subdural haemorrhage (acute)(nontraumatic)

I62.1 Nontraumatic extradural haemorrhage

I62.9 Intracranial haemorrhage (nontraumatic), unspecified

*Clotting-related admissions*

I63 Cerebral infarction

I64 Stroke, not specified as haemorrhage or infarction

I26 Pulmonary embolism

I82 Other venous embolism and thrombosis

I80.0 Phlebitis and thrombophlebitis of superficial vessels of lower extremities

I80.1 Phlebitis and thrombophlebitis of femoral vein

I80.2 Deep vein thrombosis, Phlebitis and thrombophlebitis of other deep vessels of lower extremities

I80.3 Phlebitis and thrombophlebitis of lower extremities, unspecified

I80.8 Phlebitis and thrombophlebitis of other sites

I80.9 Phlebitis and thrombophlebitis of unspecified site

**Appendix 3. Decision aids used as part of the SMOR intervention**

**3.1 – Decision aid for initiating a DOAC**

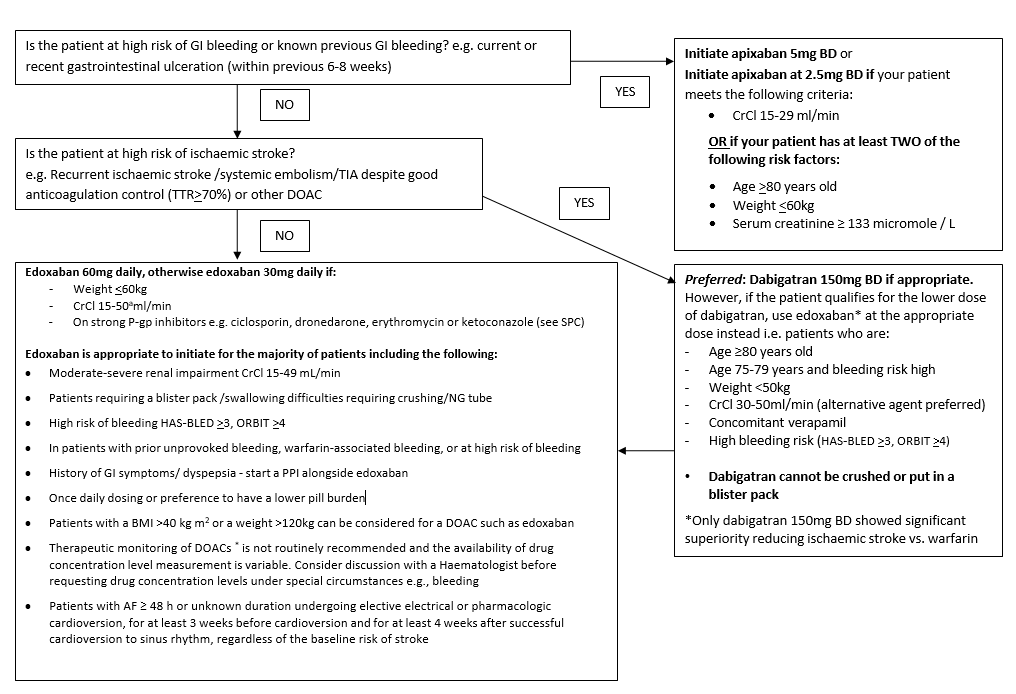


**3.2 – Decision aid for medicines optimisation review**

Table to support with decision of DOAC

| **Patient Characteristics** | **Preferred Choice DOAC Recommended** |
| --- | --- |
| Recurrent ischaemic stroke/systemic embolism/TIA despite good anticoagulation control (TTR>70%) or other DOAC | Dabigatran 150mg BD preferred if high dose appropriate |
| Patient requiring blister pack or poor swallowing requiring tablets to be crushed | Edoxaban preferred  Avoid dabigatran |
| High risk of GI bleeding e.g. current or recent gastrointestinal ulceration, requiring ongoing upper GI surveillance by a specialist | Apixaban preferred* |
| High risk of bleeding (HAS-BLED >3 /ORBIT >4) excluding gastric bleeding | Edoxaban preferred |
| Patient preference for once daily | Edoxaban preferred |
| CrCl 15-29mls/min** - Use with caution, recheck in 4 weeks (and every 3 months thereafter) | Edoxaban 30mg daily preferred |
| History of GI symptoms/ dyspepsia but no active symptoms | Start a PPI and Edoxaban preferred |
| *check body weight and age to check if patient qualifies for lower dose Apixaban  **CrCl<20mls/min consider if OAC appropriate  See ‘Initiating a DOAC Decision Aid’ for abbreviation key | |

**3.3 – Standard Operating Procedure (SOP) for Medicine Optimisation Reviews for Direct-Acting Oral Anticoagulants (DOACs) in Atrial Fibrillation (AF)**

**Applies to:**

Pharmacists and GPs working in an NHS Healthcare Trust or Place.

This protocol is produced by Cheshire and Merseyside Healthcare Partnerships (HCP).

**Aims:**

- To review all patients currently prescribed a DOAC for AF, assess whether treatment is still clinically appropriate and check the dose is correct.
- To review patients currently prescribed apixaban to determine if an alternative DOAC with a lower acquisition cost is clinically appropriate and change if safe to do so after discussion with the patient/carer.

**Background:**

Direct-acting oral anticoagulants (DOACs) or non-vitamin K antagonist oral anticoagulants (NOACs) are an alternative anticoagulant to prevent strokes in patients with Atrial Fibrillation (AF). All patients with the following should be considered for an oral anticoagulant ([NICE NG196^1^](https://www.nice.org.uk/guidance/ng196)):

- symptomatic or asymptomatic paroxysmal, persistent or permanent atrial fibrillation
- atrial flutter
- a continuing risk of arrhythmia recurrence after cardioversion back to sinus rhythm

The Cheshire and Merseyside Integrated Care Board (ICB) are supporting the review of all patients currently receiving a Direct Oral Anticoagulant (DOAC) or non-vitamin K antagonist oral anticoagulant (NOAC) for stroke prevention in AF. This project is supported by the Northwest Cardiac Strategic Clinical Network and Cardiovascular Board.

All patients on a DOAC for AF should have a medicine optimisation review to ensure their existing DOAC is appropriate according to recent bloods and weight. All patients prescribed any oral anticoagulant (OAC) should discuss the options with a healthcare professional at least once a year [NICE QS93](https://www.nice.org.uk/guidance/qs93)^2^. Adherence and compliance should be assessed regularly to support patients to take medication appropriately and safely. Stroke and bleeding risk will change over time and must be recalculated at least annually. Blood monitoring including Haemoglobin (Hb), liver and renal function should be monitored at least annually, and more regularly in people with renal dysfunction, over the age of 75 years or those who are frail. The 2018 European Heart Rhythm Association Guidelines ([EHRA 2018](https://academic.oup.com/eurheartj/article/39/16/1330/4942493)^3^) recommends monitoring as per table 1.

Currently, all DOACs (edoxaban, dabigatran, rivaroxaban and apixaban) are recommended as options for anticoagulation in the NICE AF Guidelines [(NG196^1^)](https://www.nice.org.uk/guidance/ng196) considering individual patient bleeding risks and co-morbidities. NHS England (NHSE) recently published [Commissioning Guidance](https://u4978464.ct.sendgrid.net/ls/click?upn=f3Ut4HzWYQjyfZa8pd8QHN-2FdG7ICCLVOVowUM3amfGLBD4ma7cullDKd-2F1iBLaRZTb8JkRsWIst1JEcnV-2FLqOxlK-2F8Yyn-2B2KguyNedbM3m2cZ2V-2BPM77Kpni-2F2ovfgKlIsWg-2F6hZBz2-2BRichhv17KwnjXZVOnNHx9tAhwSUShizeYM2cd2ffIci4T2MjGQVqkPP__C0iibycwa8saRXBwPDH5uJC6YA8ET1RZ-2FrNPY-2FBMR7mYOwRY5TD6i7RDlyxlzqgQwDaw8xh2-2FKYD4Ue-2FFJ1LfqrA0kxCS4zykGD58XisbikWU-2F8G7-2FCtFSleZDW9zyk1Z4oolLqRGsyeyZmYchZNZvG9iqel1J0Qb2jgNb-2F9IvtcVjNOf-2BYR34tmowCb-2BFRJZN9a6iLe390lotULHCVDog-3D-3D) ^4^ for DOACs which recommends that clinicians should use edoxaban, where clinically appropriate, consistent with the latest guidance from NICE ^1^. This approach has also been endorsed by the UK’s leading stroke charity, Stroke Association.

There are no head-to-head comparative trials that demonstrates that one DOAC is significantly better than another and treatment should be based on individual patient factors and bleeding risk. Patients newly diagnosed with AF who require anticoagulation should be initiated on edoxaban unless there is a clinical reason to use warfarin or another DOAC.

**Objectives:**

- To optimise the care of patients on a DOAC
- To identify patients in whom a DOAC is not appropriate
- To identify patients in whom the dosage of DOAC prescribed needs amending: either increasing or decreasing
- To identify patients who need (additional) monitoring
- To identify patients on interacting medication and change as appropriate
- To identify patients on concomitant antiplatelet and/or anticoagulants and adjust treatment accordingly
- To identify patients prescribed a DOAC with the current highest acquisition cost (apixaban) to review if they are clinically appropriate to change to another DOAC of a lower acquisition cost
- To provide outcomes of the reviews to the HCP for analysis. No patient identifiable data will be shared.

**Rationale:**

To ensure that all patients are on an appropriate DOAC and dose regime for their individual renal function, liver function, weight, co-morbidities and medication, to ensure optimal oral anticoagulant (OAC) therapy. Given the current significant price difference between apixaban and the other DOACs available, patients should also be reviewed to consider if an alternative DOAC of a lower acquisition cost, could be prescribed instead of apixaban.

**Inclusions:**

- All patients with AF prescribed a DOAC will be reviewed to ensure medicine optimisation of their DOAC and amended accordingly, if appropriate

All patients with AF prescribed apixaban should be reviewed to consider changing them to a DOAC of lower acquisition cost as part of the medicine's optimisation review.

**Exclusions:**

- Patients on a DOAC for another documented indication other than AF

**Responsibilities:**

To be agreed at Place level;

- Each practice should determine whether a pharmacist or GP will undertake the optimisation reviews of DOACs. The method has been written as per a pharmacist review.
- Pharmacy technicians could be responsible for running the EMIS search and report, as per protocol and highlighting patients with any outstanding monitoring to the pharmacist or GP.
- The pharmacist or GP is responsible for undertaking the reviews, as per protocol and training provided.
- The pharmacist or GP undertaking the review is responsible for ensuring the patient has given verbal consent, which is documented in the PMR, if further advice needs to be sought from a specialist external to the GP practice.
- GP Practice to agree for non-identifiable patient information to be collated for service evaluation at a regional level.
- The GP is responsible for agreeing for the work to be carried out in the practice, ensuring all monitoring and values are up to date and for following up any patients identified during the project that require further review.

**Method:**

| **Action** | **Who is responsible** |
| --- | --- |
| **DOAC Review** | |
| 1. Gain agreement from the GP practice to run the EMIS search and report and to review all patients with NV-AF prescribed DOACs, as per protocol. Gain agreement from the GP practice to review patients prescribed apixaban, to determine if an alternative DOAC with a lower acquisition cost is clinically appropriate, then change. Agree with GP practice action to be taken if outstanding monitoring is identified. | **Pharmacist / GP** |
| 1. Notify the Local Pharmaceutical Committee (LPC) and GP practice staff of work being undertaken. | **Pharmacist/Technician** |
| 1. Run the approved EMIS search and reports to identify all patients in the practice on existing DOAC therapy – edoxaban, apixaban, rivaroxaban or dabigatran who also have a code for AF or atrial flutter. | **Technician** |
| 1. Check EMIS report to see if monitoring of U+E’s, FBC, LFTs, BP*, HR* and actual body weight are within the preceding 3 months and if not, highlight any required monitoring to the GP practice or action as agreed by the GP practice.   Review each patient once all values** have been received and documented in the patient’s medical record (PMR).  Each practice should consider how best to recall patients for monitoring. Factors to consider include:   - Number of patients - How and when the medicines management team will check that monitoring is complete - How to update the patient list as patients join and leave the practice   ***NB: consider if HR >100 bpm, refer to GP for rate control. BP consistently above 140/90 should be discussed with GP or reviewed according to local guidelines.**  ****NB: as per local reference ranges** | **Pharmacist/Technician** |
| 1. The EMIS report highlights patients with the following:    - Possible valvular heart disease    - Possible unlicensed indications for DOAC treatment    - History of thromboembolism    - Two oral anticoagulants on current medication   **NB. If you identify that a patient is currently taking a DOAC for an indication other than AF it is good practice to confirm the dose and duration of treatment is appropriate.** | **Technician** |
| **In consultation, run the EMIS DOAC Review Template** | |
| 1. Calculate and update the CHA2DS2-VASc^8^ score using the EMIS calculator     **NB: Patients with a CHA2DS2-VASc =1 in men or =2 in women should**  **be considered for an oral anticoagulant (OAC).**  **Patients with a CHA2DS2-VASc score >2 in men and >3 in women:**  **It is recommended that these patients should be prescribed an OAC.** | **Pharmacist** |
| 1. Calculate and update the HAS-BLED score to assess the risk of bleeding in people on anticoagulation. NICE [NG196](https://www.nice.org.uk/guidance/ng196)^1^ AF Guidelines recommends using the ORBIT score to calculate the risk of bleeding. Until this is embedded into EMIS it is acceptable to continue using the HAS-BLED score or calculate both scores to make an informed decision (see below).   Refer to GP if clarification needed for modification, monitoring or advice, if there is no documentation that the following risk factors have been considered:   - Uncontrolled hypertension - Concurrent medication that will increase bleeding risk; - Anti-platelet medication e.g. aspirin - Non-steroidal anti-inflammatory drugs (NSAIDs) - SSRI’s or SNRI’s - Harmful alcohol consumption above national recommendations (if noted on PMR)   Calculate the ORBIT score^9^  Offer monitoring and support to modify risk factors for bleeding, including:   - uncontrolled hypertension (see NICE's guideline [NG136](https://www.nice.org.uk/guidance/ng136)^10^ on hypertension in adults) - concurrent medication, including antiplatelets, selective serotonin reuptake inhibitors (SSRIs) and non-steroidal anti-inflammatory drugs (NSAIDs) - harmful alcohol consumption (see NICE's guideline [CG115](https://www.nice.org.uk/guidance/cg115)^11^ on alcohol-use disorders: diagnosis, assessment and management of harmful drinking and alcohol dependence) - reversible causes of anaemia.   With patient permission, Secondary Care advice with a Specialist Pharmacist may be sought via email if there are additional queries or concerns that the pharmacist or GP is unable to resolve. If no local specialists are available advice may be requested from xxxxxxxxx, Project Lead and Chair of Pharmacist Cardiac and Stroke Forum, Northwest Strategic Clinical Network. Queries will be escalated for consultant advice if required.  Particularly complicated patients can be referred to the AF Specialist Clinic at Liverpool Heart and Chest Hospital (LHCH) or the Clinical Haematology Service, Roald Dahl Haemostasis and Thrombosis Centre, Liverpool University Hospitals NHSFT for advice about anticoagulation.  **Consider:**  A high bleeding risk score should generally not result in withholding OAC. Rather, bleeding risk factors should be identified, and treatable factors corrected. If patients are on concomitant anti-platelets, review if these are appropriate. Consider stopping if >1 year post-acute coronary syndrome (ACS) or stable coronary artery disease. Discuss with GP/Specialist if necessary.  Consider gastro-protection with appropriate concomitant anti-platelets. | **Pharmacist** |
| 1. Calculate the Creatinine Clearance (CrCl)  - All DOACs may require a dose adjustment based on renal impairment - **Creatinine clearance must be used for calculating renal function** using the Cockcroft and Gault equation (see below). eGFR is **not** a suitable alternative:   CrCl (ml/min )= (140 – age) x wt (kg) x 1.04 (female) or 1.23 (male)  serum creatinine (micromol/l)   - The actual body weight must be used to calculate CrCl   **NB The EMIS clinical system used in primary care has an inbuilt Cockcroft-Gault based renal function calculator which can be used to dose DOACs. EMIS recognises if the patient is prescribed a DOAC and will use actual body weight to calculate CrCl for these patients. The exception are patients taking Dabigatran who are also obese, in this case the calculator uses ideal body weight. In these cases, you should record the creatinine clearance for actual body weight in the consultation. Actual body weight was used in AF trials for all DOACs.**   - Another option is to use the MD+ CALC Creatinine Clearance calculator (it can be downloaded as an app to an apple or android device). Always use the most up to date values and check the default units are correct when entering weight and serum creatinine. It would be good practice for the clinician reviewing the patient to document what method was used - Document the method used to calculate CrCl on the template   **NB:**   - **Pts with CrCl < 15mls/min – refer to GP/Specialist for review as contraindicated (if CrCl borderline 15 – 20mls/min this should be discussed with GP to ensure a DOAC is still appropriate)** - **Pts on Dabigatran and CrCl<30mls/min – contraindicated and refer to GP/Specialist for alternative DOAC** | **Pharmacist** |
| 1. Confirm if the current DOAC dose and indication is correct and the current DOAC dose is appropriate for patients with AF, according to current parameters – see SPC of each DOAC. | **Pharmacist** |
| 1. Check all medication for any significant drug interactions including hospital prescribed medication, OTC and herbal/alternative therapy– see SPCs for full details.   **NB: If prescribed anti-platelets, consider whether these need to be continued and if unsure, discuss with Specialist, as per point 7.** | **Pharmacist** |
| 1. Discuss any patients on incorrect dose/significant interactions/safety concerns with GP.   NB:  Where the efficacy of either the DOAC or another medication is affected please ensure this is discussed with the GP and/or relevant specialist e.g. antiepileptics | **Pharmacist** |
| 1. Following agreement by the GP practice, patients on apixaban should be reviewed as per the Decision Aid for Medicines Optimisation Review of Patients Prescribed Apixaban, to consider if an alternative DOAC is clinically appropriate | **Pharmacist** |
| 1. Discuss any other identified issues or queries with the GP as appropriate and document the outcome. | **Pharmacist** |
| 1. Contact the patient to review adherence, side effects, adverse drug reactions (ADRs) or any issues raised during the review. Discuss and agree with patient any recommended changes | **Pharmacist** |
| **Patient review and informed discussion via phone or virtual consultation** | |
| 1. Contact the patient/carer as agreed by the practice (telephone or virtual) and go through the patient section of the EMIS DOAC template, checking for any;  - GI symptoms (consider addition of PPI or discuss with GP) - Swallowing difficulties - Bleeding - OTC or herbal medications - Adherence - Adverse drug reactions - Side-effects - Excessive alcohol intake - Re-calculate HASBLED/ORBIT score if necessary - Check if patient uses a monitored dosage system and inform community pharmacy of any changes.   Use the checklist to ensure you cover all the relevant counselling | **Pharmacist** |
| 1. Advise patients of the issues identified e.g. DOAC agent to be changed or dose change and make the appropriate changes or if there are any interactions that patients need to be aware of | **Pharmacist** |
| 1. For any concerns or further issues identified – discuss with the GP or Specialist and document the outcome. Advise patient that you will inform them of outcome of discussion with GP or Specialist. | **Pharmacist** |
| 1. Use a suitable code such as ’*Medication Review done by Medicines Management Pharmacist’** and set an appropriate follow up review date that considers the criteria in Table 1.   ***NB: local agreement may differ where and how to record on PMR** | **Pharmacist** |
| 1. Document the outcomes of the DOAC review on the final outcomes report, as agreed at Place level*   ***No patient identifiable data should be collected on the outcome report** | **Pharmacist** |

**3.3 Frequently Asked Questions (FAQs)**

[FAQs document](https://theuniversityofliverpool.sharepoint.com/sites/hiprt/Shared%20Documents/Hip-R%20Projects/CIPHA_systemP/DOACs/paper/UPDATED%20Cheshire%20and%20Mersey%20FAQ%20-%20Medicines%20Optimisation%20of%20DOACs%20in%20AF%20Sept%2022.pdf)

**Appendix 4. Cumulative proportion of people with AF on a DOAC undergoing a structured medication optimisation review (SMOR) in Liverpool and Sefton 2021-2023.**

**
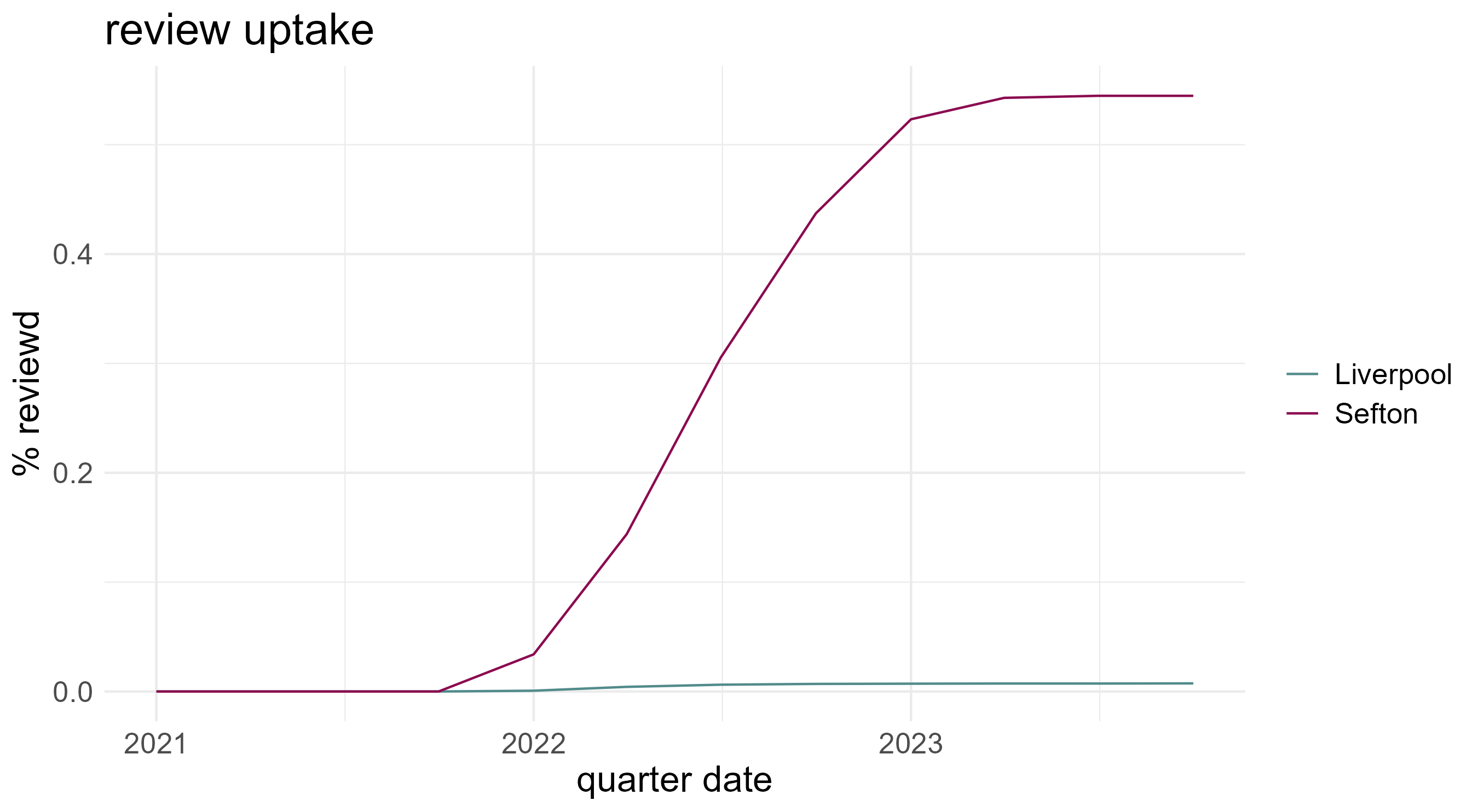
**

**Appendix 5. Codes used to define the structured medication optimisation review (SMOR).**

We defined a review as having taken place if a patient in Sefton had been coded with either DOAC dose changed (SNOMED 1364161000000108) or DOAC dose unchanged (SNOMED 1364181000000104) and had a record of bleeding risk check (SNOMED 1791201000000119 or 790721000000110).

**Appendix 6. Change in bleeding related admissions (per 1000 patients reviewed per year) attributable to the intervention estimated from the doubly robust difference in differences model: sensitivity analyses.**

| Model | Estimate | 95% CI | |
| --- | --- | --- | --- |
| Base case model (controls from both Sefton and Liverpool, inverse probability tilted model) | -8.1 | -22.2 | 6.1 |
| Controls just from Sefton | -8.76 | -23.4 | 5.88 |
| Controls just from Liverpool | -6.76 | -21.2 | 7.72 |
| Inverse probability weighted model | -7.88 | -22.2 | 6.48 |
